# Are Japanese Women Less Physically Active Than Men? Findings From the DOSANCO Health Study

**DOI:** 10.2188/jea.JE20200185

**Published:** 2021-10-05

**Authors:** Shiho Amagasa, Shigeru Inoue, Shigekazu Ukawa, Sachiko Sasaki, Koshi Nakamura, Aya Yoshimura, Aya Tanaka, Takashi Kimura, Takafumi Nakagawa, Akihiro Imae, Ding Ding, Hiroyuki Kikuchi, Akiko Tamakoshi

**Affiliations:** 1 Department of Preventive Medicine and Public Health, Tokyo Medical University, Tokyo, Japan; 2 Research Unit of Advanced Interdisciplinary Care Science, Osaka City University Graduate School of Human Life Science, Osaka, Japan; 3 Department of Public Health, Faculty of Medicine, Hokkaido University, Hokkaido, Japan; 4 Department of Physical Therapy, Faculty of Human Science, Hokkaido Bunkyo University, Hokkaido, Japan; 5 Department of Public Health and Hygiene, Graduate School of Medicine, University of the Ryukyus, Okinawa, Japan; 6 The Hokkaido Centre for Family Medicine, Hokkaido, Japan; 7 Suttsu Municipal Clinic, Hokkaido, Japan; 8 Prevention Research Collaboration, Sydney School of Public Health, University of Sydney, Sydney, Australia

**Keywords:** accelerometry, exercise, sedentary lifestyle, middle-aged, physical activity

## Abstract

**Background:**

Previous research has established that women accumulate less moderate-to-vigorous physical activity (MVPA) than men. To date, however, little is known about the gender differences in device-based activity patterns of sedentary behavior (SB) and light-intensity physical activity (LPA). We aimed to compare time spent in SB and different intensities of physical activity taking into account of co-dependence of time use domains.

**Methods:**

This cross-sectional study was conducted in Suttu town, Hokkaido, Japan. Data were analyzed from 634 Japanese adults (278 men, aged 19–92 years) who provided valid accelerometer (HJA-750C) data. Gender differences in activity behavior patterns were tested using multivariate analysis of covariance (MANCOVA) based on isometric log-ratio transformations of time use, adjusting for age. We also developed bootstrap percentile confidence intervals (CI) to support the interpretation of which behavior differed between genders.

**Results:**

Overall, participants had percent time spent in SB, LPA, MVPA during wearing time (mean, 14.8 hours) corresponding to 53.9%, 41.7%, and 4.4% of wearing time, respectively. Activity behavior patterns differed significantly between genders after controlling for time spent in all activities. Women spent relatively 13.3% (95% CI, 9.9–15.9%) less time in SB and 19.8% (95% CI, 14.9–24.6%) more time in LPA compared to men. The difference of time spent in MVPA was not statistically significant.

**Conclusions:**

In contrast with previous studies, our findings suggest that Japanese women are more physically active than men when all intensities of activities are considered. Given the health benefits of LPA, evaluating only MVPA may disproportionately underestimate the level of physical activity of women.

## INTRODUCTION

Evidence from global surveillance of physical activity repeatedly identified women to be less physically active than men in almost every country, when physical activity was measured by adherence to guidelines.^1^^–^^3^ Based on the most recent statistics, the global prevalence of insufficient physical activity was estimated to be 23.4% in men and 31.7% in women.^3^ Most of these studies defined physical activity according to global physical activity guidelines, which recommend that adults engage in at least 150 minutes of moderate-to-vigorous physical activity (MVPA) per week in bouts lasting at least 10 minutes.^4^ However, bouted activities constitutes a small proportion of one’s weekly time.^5^^–^^7^

In recent years, accelerometers have become commonly used in research, which has allowed for examination of unbouted or shorter bouts of physical activity. A majority of physical activity research have relied on self-report, so we could not look closely into bouts.^8^ A recent systematic review found that physical activity of any bout duration was associated with improved health outcomes.^9^ For example, a study found that the overall time spent in MVPA, rather than how MVPA was accumulated, was associated with risk reduction of all-cause mortality.^7^ Further, recent evidence also suggests the detrimental effects of sedentary behavior (SB)^10^^–^^13^ and beneficial effects of light-intensity physical activity (LPA) on health.^5^^,^^6^^,^^14^ For example, a meta-analysis of device-based measurement studies found that replacing SB time with LPA was favorably associated with all-cause mortality risk and cardiometabolic risk markers.^13^ Therefore, there is a need to examine physical activity across the intensity spectrum, bouted or unbouted.^8^

Our recent study for community-dwelling older Japanese adults has shown that, contrary to the existing evidence,^1^^–^^3^ when taking into account of physical activity without bouts, the level of physical activity among women was actually greater than men, owing to longer time spent in LPA.^15^ However, the generalizability of previous findings to middle-aged adults remains unclear. Moreover, in our previous study, the co-dependence of time-use domains was not totally taken into account. Recent developments of compositional data analysis (CoDa) allows for consideration of the co-dependence of time spent in activities within a day,^16^^–^^18^ providing a more comprehensive understanding of the overall patterns of physical activity. In the current study, we aimed to compare men and women’s time spent in physical activity-related behaviors, while taking into consideration of the co-dependence of time use domains. We hypothesized that men accumulated more MVPA and SB, whereas women accumulated more LPA.

## METHODS

### Study sample and data collection

This cross-sectional study was a part of the Dynamics of Lifestyle and Neighborhood Community on Health Study (DOSANCO Health Study), a population-based survey conducted in Suttu town, Hokkaido, Japan, in 2015.^19^ Suttu town is small rural area (area: 95.3 km^2^, population: 3,259, as of December 31, 2014). Briefly, a total of 2,638 residents (all residents) who were aged 3 years or older and not in nursing homes were targeted and 2,100 participants responded to a questionnaire (children [3–17 years], *n* = 205; adults [18–64 years], *n* = 1,083 [men: 550, women: 533]; older adults [≥65 years], *n* = 812 [men: 324, women: 488]). Of these, in the summer and autumn of 2015, 808 participants took health examination survey, and at the same time they were asked to enroll in accelerometer survey. In the end, 771 participants (children, *n* = 84; adults, *n* = 412 [men: 192, women: 220]; older adults, *n* = 275 [men: 114, women: 161]) agreed to wear an accelerometer (response rate 29.2%). The University Ethics Committee (Hokkaido University and Tokyo Medical University) granted ethical approval. Informed consent was obtained from all participants prior to the survey.

### Measurement of activity behavior patterns

Participants were instructed to wear an accelerometer, the Active style Pro HJA-750C (Omron Healthcare, Kyoto, Japan), for 14 consecutive days while awake, except during water-based activities (eg, swimming). Active style Pro is a validated accelerometer^20^^–^^23^ that provides data comparable to the most commonly used devices in studies conducted in Western countries.^24^^,^^25^ Its measurement algorithm has been explained in detail elsewhere.^20^^,^^21^ No detected acceleration signal for longer than 60 consecutive minutes was defined as “non-wear”, and records from participants wearing the accelerometer for at least 10 hours per day were considered valid.^26^ Participants with 4 or more valid wear days were included in the analyses.^27^^,^^28^ The mean wear time and time spent in each activity on valid days was used for the analysis. We used a standard 60-second epoch data to allow for comparison with previous studies.^29^^,^^30^ Metabolic Equivalents (METs)-based criteria was used to determine intensity of activities: ≤1.5 METs for SB, 1.6–2.9 METs for LPA, and ≥3.0 METs for MVPA.^31^^,^^32^ Consistent with previous research, 10-minute bouts of MVPA were defined as 10 or more consecutive minutes above the moderate intensity threshold, with allowance for interruptions of 1–2 min per 10 minutes below the threshold.^28^^,^^33^ MVPA lasting 8 or 9 minutes without interruptions was not defined as 10-min bouts. The protocol applies to all sub-compositions of activities that constitute accelerometer wearing time (SB, LPA, and MVPA).

### Sociodemographic, biological, and psychological factors

Participants reported their age, gender, living arrangement (with others/alone), working status (workers/non-workers), and perceived health. Perceived health was assessed using one question that asked participants to rate their health on a 4-point scale: very good, good, poor, and very poor. The answers were further categorized into “good” (very good/good) and “poor” (poor/very poor). Weight were measured using InBody430 (InBody Japan, Tokyo, Japan). Body mass index (BMI) was calculated from height and weight (kg/m^2^).

### Statistical analyses

The proportions of those who adhered to the global physical guidelines (≥150 minutes/week of 10-min bout MVPA) and the physical activity guidelines for Japanese adults (≥23 METs-hour/week of unbouted MVPA) were calculated. The chi-square test, *t*-test, multivariate analysis of variance or analysis of covariance was performed to compare participant characteristics between genders. Ternary diagram was used to illustrate the sample compositions of time spent in each activity. We used CoDa approach as detailed in previous research.^16^^,^^34^ Variability in the data, in terms of variability of each behavior relative to the variability of other activities was described through a variation matrix.^16^^,^^35^ No statistical method was required to impute zero since all participants spent some time in each behavior.

Time spent in SB, LPA, and MVPA was transformed into isometric log-ratio (ilr) coordinates. Since we use a three-part composition (SB, LIPA, and MVPA), each movement behavior is then represented by two ilr variables z_1_ and z_2_. Ilr-coordinate z_1_ represents the relative importance of one component (eg, MVPA) relative to the geometric mean of the other components (eg, SB and LPA). For instance, MVPA relative to SB and LPA is isolated as:

$$z_{1}=\sqrt{\frac{2}{3}}ln\frac{MVPA}{\sqrt[{2}]{SB\times LPA}}$$

$$z_{2}=\sqrt{\frac{1}{2}}ln\frac{SB}{LPA}$$

We also isolated SB or LPA relative to the other components. Therefore, a total of six ilr variables were made with pair of two variables (eg, z_1_ and z_2_) for each component (SB, LPA, and MVPA).

The multivariate analysis of covariance (MANCOVA) was used to test whether the activity compositions differed between men and women after adjustment for sociodemographic factors. Models were adjusted for age (model 1) and age, living arrangement, and working status (model 2). To further support the interpretation of which behavior is significant group difference, we developed bootstrap percentile confidence intervals (CI) for log-ratio differences between genders.^34^^,^^36^ We created 10,000 virtual datasets for bootstrap. First, we analyzed the whole sample and then stratified by age group (19–64 and ≥65 years). We performed sensitivity analyses with different criteria for the number of valid wearing days (7 days, 10 days, and 14 days). R version 3.5.2 (R Foundation for Statistical Computing, Vienna, Austria) was used to perform all statistical analyses. Statistical significance was set at *P* < 0.05.

## RESULTS

### Participant enrollment and descriptive statistics

Of the 687 adults who returned an accelerometer, 53 were excluded for not meeting accelerometer wearing time criteria. Thus, the final analytic sample was 634 in this study. No significant differences of accelerometry respondents were found in gender (men: 35.6%, women: 38.0%).

**Figure 1.**
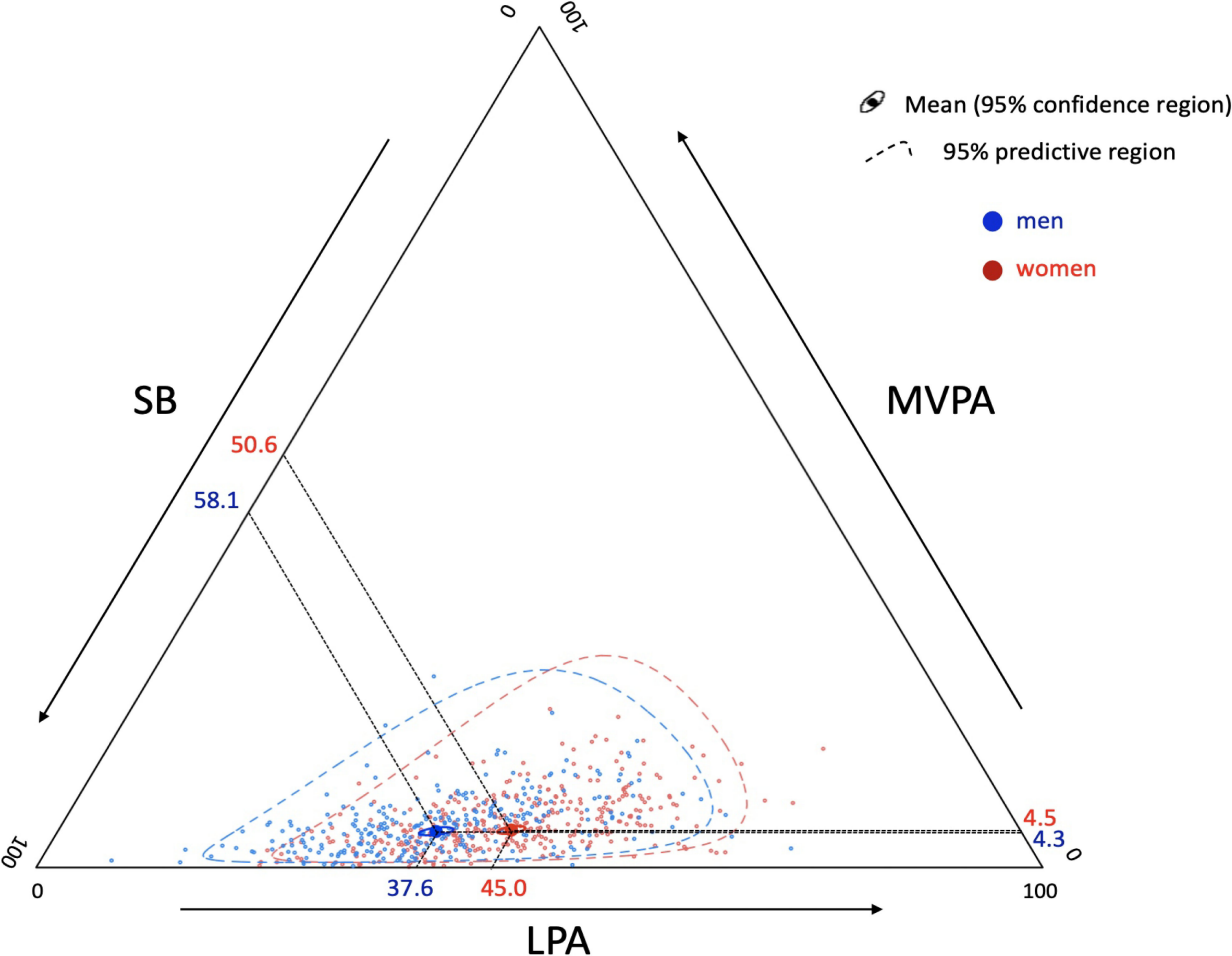
Ternary diagrams of the sample compositions of time spent in sedentary behavior, light-intensity physical activity, and moderate-to-vigorous physical activity. Activity computations differed significantly between genders (multivariate analysis of variance, *P* < 0.001). LPA, light-intensity physical activity; MVPA, moderate-to-vigorous physical activity; SB, sedentary behavior.

Table 1 presents the characteristics of the participants. Overall, the mean age was 57.9 (standard deviation [SD], 16.9) years and mean value of accelerometer wear time was 873.4 (SD, 91.6) minutes/day. Participants spent 464.5 (SD, 114.5) min/day in SB, 361.5 (SD, 96.2) min/day in LPA, 47.1 (SD, 30.6) min/day in MVPA. MVPA consist mostly of MVPA lasting <10 minutes (men: 85.1%, women: 87.3%). Compared to men, women were significantly more likely to be non-workers. There were no significant gender differences in the proportion of those adhering to global physical activity guidelines (men: 10.8%, women: 9.9%) and daily step counts (men: 4,899 steps/day, women: 4,580 steps/day). Women significantly accumulated greater volume of physical activity than men (men: 14.0 METs-hour/day, women: 16.1 METs-hour/day). Activity behavior patterns differed significantly between genders (Figure 1).

**Table 1.** Characteristics of study participants and time spent in sedentary behavior and physical activity by gender

	Men (*n* = 278)	Women (*n* = 356)	*P*-value

	*n* (%)/mean (SD or 95% CI)	*n* (%)/mean (SD or 95% CI)	
Age, years	56.7 (17.2)	58.9 (16.7)	0.105^b^
Working status, working	203 (73.6%)	197 (55.5%)	**0.001** ^a^
Living arrangement, with others	221 (79.5%)	281 (78.9%)	0.862^a^
Body mass index, kg/m^2^	24.1 (3.5)	23.7 (3.9)	**0.005** ^b^
Perceived health, good	211 (80.2%)	276 (80.0%)	0.944^a^
WHO physical activity guidelines,^c^ meeting	30 (10.8%)	35 (9.9%)	0.701^a^
Total volume of physical activity, METs-hour/day	14.0 (13.5, 14.5)	16.1 (16.5, 17.0)	**<0.001** ^d^
Step count, steps/day	4,899 (4,646, 5,151)	4,580 (4,357, 4,803)	0.065^d^
Accelerometer wear time, min/day	862.2 (94.6)	882.1 (88.3)	**0.007** ^b^
Standard analysis, arithmetic mean			
SB, min/day	494.0 (118.5)	441.5 (105.9)	**<0.001** ^b^
LPA, min/day	322.91 (88.9)	391.6 (90.9)	**<0.001** ^b^
MVPA, min/day	45.2 (29.4)	48.6 (31.4)	0.155^b^

CI, confidence interval; LPA, light-intensity physical activity; MVPA, moderate-to-vigorous physical activity; SB, sedentary behavior; SD, standard deviation.

Missing value: working status *n* = 3, perceived health *n* = 26.

^a^chi-squared test.

^b^*t*-test.

^c^150 min/week of moderate-to-vigorous physical activity in bouts of at least 10 minutes.

^d^Analysis of covariance (adjusted by age and wear time).

Table 2 shows the variation matrix indicating the dispersion of each behavior. The highest log-ratio variances all involved MVPA, which indicated that time spent in MVPA was the least co-dependent on the other behaviors. The largest variability was observed in ratio of MVPA to SB, particularly in men.

**Table 2.** Variation matrix of time spent in sedentary behavior and physical activity

	SB	LPA	MVPA
Men			
SB	0		
LPA	0.228	0	
MVPA	0.711	0.313	0
Women			
SB	0		
LPA	0.195	0	
MVPA	0.691	0.356	0

LPA, light-intensity physical activity; MVPA, moderate-to-vigorous physical activity; SB, sedentary behavior.

A value close to zero implies that the times spent in the two behaviors involved in the ratio are highly proportional.

The MANCOVA test showed proportion of time spent in SB and LPA relative to the other behaviors were statistically significantly differed between men and women whereas relative proportion of MVPA was not (Table 3). After allowing for MVPA, the ratio between SB and LPA was significantly differed between genders. Additional adjustment for working status and living arrangement did not change the results. Bootstrap estimated women spent relatively 13.3% (95% CI, 9.9–15.9%) less time in SB and 19.8% (95% CI, 14.9–24.6%) more time in LPA compared to men (Figure 1). The difference of time spent in MVPA was not statistically significant (mean difference 3.2%; 95% CI, −8.0 to 17.2%).

**Table 3.** Results of multivariate analysis of variance of differences in sedentary and physically-activity time

Independent variable	Dependent variables	Model 1 (*n* = 634)				Model 2 (*n* = 631)			

		df	Sum sq	F-value	*P*-value	df	Sum sq	F-value	*P*-value
Gender									
	MVPA/SB·LPA	1	0.356	0.985	0.321	1	0.471	1.379	0.241
	SB/LPA	1	7.909	71.905	**<0.001**	1	8.112	75.491	**<0.001**

	LPA/SB·MVPA	1	4.568	49.270	**<0.001**	1	4.509	49.779	**<0.001**
	SB/MVPA	1	3.696	9.771	**0.002**	1	4.074	11.370	**<0.001**

	SB/LPA·MVPA	1	7.473	29.563	**<0.001**	1	7.894	32.716	**<0.001**
	LPA/MVPA	1	0.792	3.627	0.057	1	0.689	3.318	0.069

LPA, light-intensity physical activity; MVPA, moderate-to-vigorous physical activity; SB, sedentary behavior.

Model 1: adjusted for age. Model 2: adjusted for age, working status, and living arrangement.

After stratified by age group, similar gender differences of time spent in activity behavior patterns were observed in adults and older adults (Table 4, Table 5, and Table 6). In addition, these results did not change even if we changed for eligible criteria for wearing days.

**Table 4.** Characteristics of study participants and time spent in sedentary behavior and physical activity by gender

	Adults (19–64 years)			Older adults (≥65 years)		

	Men (*n* = 177)	Women (*n* = 206)	*P*-value	Men (*n* = 101)	Women (*n* = 150)	*P*-value
	
	*n* (%)/mean (SD or 95% CI)	*n* (%)/mean (SD or 95% CI)		*n* (%)/mean (SD or 95% CI)	*n* (%)/mean (SD or 95% CI)	
Age, years	46.8 (12.9)	47.5 (11.8)	0.566^b^	74.1 (7.0)	74.6 (6.7)	0.597^b^
Working status, working	159 (90.3%)	161 (78.2%)	**0.001** ^a^	44 (44.0%)	36 (24.2%)	**0.001** ^a^
Living arrangement, with others	136 (76.8%)	180 (87.4%)	**0.007** ^a^	85 (84.2%)	101 (67.3%)	**0.003** ^a^
Body mass index, kg/m^2^	24.2 (3.6)	22.7 (3.9)	**<0.001** ^b^	23.8 (3.3)	23.9 (3.7)	0.859^b^
Perceived health, good	141 (81.5%)	176 (86.3%)	0.207^a^	70 (77.8%)	100 (70.9%)	0.249^a^
WHO physical activity guidelines,^*^ meeting	22 (12.4%)	25 (12.1%)	0.930^a^	8 (7.9%)	10 (6.7%)	0.706^a^
Physical activity guidelines for Japanese,^†^ meeting	64 (36.2%)	85 (41.3%)	0.307^a^	N/A		
Total volume of physical activity, METs-hour/day	14.4 (13.8, 15.0)	17.4 (16.9, 18.0)	**<0.001** ^c^	13.5 (12.7, 14.3)	15.2 (14.6, 15.9)	**0.002** ^c^
Step count, steps/day	5,614 (5,291, 5,938)	5,438 (5,138, 5,738)	0.434^c^	3,769 (3,386, 4,152)	3,318 (3,005, 3,632)	0.075^c^
Accelerometer wear time, min/day	874.4 (93.5)	897.2 (85.2)	**0.014** ^b^	840.7 (92.9)	861.2 (88.5)	0.079^b^
Standard analysis, arithmetic mean						
SB, min/day	501.0 (118.4)	436.1 (101.3)	**<0.001** ^b^	481.6 (118.2)	449.0 (111.7)	**0.027** ^b^
LPA, min/day	321.1 (81.8)	403.9 (83.9)	**<0.001** ^b^	326.0 (100.5)	374.8 (97.6)	**<0.001** ^b^
MVPA, min/day	52.1 (30.1)	56.8 (30.7)	0.128^b^	33.0 (23.9)	37.4 (28.9)	0.209^b^
CoDa, geometric mean						
SB, % of wear time	57.8	48.8	**<0.001** ^d^	58.4	52.8	**<0.001** ^d^
LPA, % of wear time	36.9	45.5		38.7	44.0	
MVPA, % of wear time	5.3	5.7		3.0	3.2	

CI, confidence interval; CoDa, compositional data analysis; LPA, light-intensity physical activity; MVPA, moderate-to-vigorous physical activity; SD, standard deviation; SB, sedentary behavior.

^*^150 minutes/week of moderate-to-vigorous physical activity in bouts of at least 10 minutes.

^†^23 METs-hour/week of unbouted moderate-to-vigorous physical activity.

^a^chi-squared test.

^b^*t*-test.

^c^Analysis of covariance (adjusted by age and wear time).

^d^Multivariate analysis of variance (MANOVA).

Missing value: working status *n* = 3, perceived health *n* = 26.

**Table 5.** Variation matrix of time spent in sedentary behavior and different intensities of physical activity

	Adults (19–64 years)			Older adults (≥65 years)		

	SB	LPA	MVPA	SB	LPA	MVPA
Men						
SB	0			0		
LPA	0.185	0		0.289	0	
MVPA	0.544	0.195	0	1.306	0.608	0
Women						
SB	0			0		
LPA	0.171	0		0.190	0	
MVPA	0.445	0.232	0	0.980	0.578	0

SB, sedentary behavior; LPA, light-intensity physical activity; MVPA, moderate-to-vigorous physical activity.

A value close to zero implies that the times spent in the two behaviors involved in the ratio are highly proportional.

**Table 6.** Results of multivariate analysis of variance of differences in sedentary and physically-activity time

Independent variable	Dependent variables	Adults (19–64 years)								Older adults (≥65 years)							
	
		Model 1 (*n* = 383)				Model 2 (*n* = 382)				Model 1 (*n* = 251)				Model 2 (*n* = 249)			

		df	Sum sq	F-value	*P*-value	df	Sum sq	F-value	*P*-value	df	Sum sq	F-value	*P*-value	df	Sum sq	F-value	*P*-value
Gender																	
	MVPA/SB·LPA	1	0.212	0.888	0.347	1	0.313	1.322	0.251	1	0.225	0.472	0.493	1	0.370	0.811	0.369
	SB/LPA	1	**6.720**	**73.159**	**<0.001**	1	**6.201**	**68.175**	**<0.001**	1	**1.635**	**12.104**	**0.001**	1	**1.733**	**13.082**	**<0.001**

	LPA/SB·MVPA	1	**4.058**	**60.113**	**<0.001**	1	**3.523**	**52.395**	**<0.001**	1	**0.758**	**6.234**	**0.013**	1	**0.699**	**5.971**	**0.015**
	SB/MVPA	1	**2.874**	**10.904**	**0.001**	1	**2.991**	**11.490**	**<0.001**	1	1.103	2.249	0.135	1	1.404	2.978	0.086

	SB/LPA·MVPA	1	**6.128**	**32.268**	**<0.001**	1	**5.935**	**31.655**	**<0.001**	1	**1.808**	**5.658**	**0.018**	1	**2.086**	**6.734**	**0.010**
	LPA/MVPA	1	**0.804**	**5.697**	**0.018**	1	**0.579**	**4.132**	**0.043**	1	0.052	0.179	0.673	1	0.017	0.062	0.804

LPA, light-intensity physical activity; MVPA, moderate-to-vigorous physical activity; SB, sedentary behavior.

Model 1: adjusted for age. Model 2: adjusted for age, working status, and living arrangement.

## DISCUSSION

The current study compared accelerometer-based time spent in activity behavior patterns between genders using a novel statistical approach. Compared to men, women had less time spent in SB and more time spent in LPA, whereas MVPA was not significantly different after controlling for time spent in all activity measures. We extended the findings from our previous analysis,^15^ which showed women are more physically active than men when all intensities of activities are evaluated.

This gender difference in activity behavior patterns could be a result of gender roles. In Japan, women have traditionally been more responsible for most of the housework. Social norms such as “Sekentei” may lead women to stay at home and engage in housework and child rearing, and thus accumulate more LPA.^37^^,^^38^ According to the National Survey on Household Changes conducted in 2018 by the National Institute of Population and Social Security Research, wives spend, on average, seven times as much time doing housework as their husbands in weekdays (263 min/day vs 37 min/day).^39^ The survey also found women still have the greater burden of housework even when the number of working women is increasing.^39^ Our findings that women engage in more LPA are consistent with those of previous studies in western countries,^40^ but the degree of gender difference is larger in Japanese population.

In this study, there was no significant gender difference in the proportion of those adhering to global physical activity guidelines and physical activity guidelines for Japanese. Japanese guidelines recommend that adults should accumulate at least 23 METs-hour/week of MVPA, which is estimated to be more than twice the volume of activity in the global recommendation of 150 minutes/week of MVPA.^41^ However, in this population, Japanese guidelines were easier to achieve than global guidelines because the Japanese guidelines did not require MVPA to be of 10-min bouts or longer. This is in line with previous findings that indicate overall MVPA consist mostly of MVPA lasting <10 minutes.^15^^,^^33^^,^^42^ Also, it is observed that people in rural (low walkable) area may accumulate less 10-min MVPA than those in urban and suburban (high walkable) area.^43^

Findings from our study indicates that the current evidence on men being more physically active than women, based primarily on bouted MVPA data, may need to be reexamined with consideration of LPA and activities of shorter bouts. Recent studies have shown that LPA is favorably associated with all-cause mortality risk and cardiometabolic biomarkers after adjustment for MVPA.^5^^,^^14^ Given health benefits of LPA, evaluating only MVPA may underestimate the level of physical activity, particularly in those who spend longer time in LPA such as women.

With regard to step count, participants in this study had lower step counts than the national average obtained from National Health and Nutrition Survey Japan (NHNSJ).^44^^,^^45^ There are several potential explanations. First, previous research in Japan showed that, on average, people living in smaller cities took fewer steps than those living in larger cities.^44^ Second, the accelerometer used in this study is more likely to underestimate the number of steps than the pedometer used in the NHNSJ (AS-200, Yamasa Co. Ltd., Tokyo, Japan).^46^ Third, the NHNSJ is conducted in the fall (November), when the number of steps is the highest of the year,^47^ so the step count is likely to be systematically higher. In terms of gender differences, in this study, there were no significant differences in daily step counts regardless of age group. This is in line with the previous evidence that rural residents tend to have smaller gender differences in step counts than residents in (sub)urban area.^44^

### Strengths and limitations

We have replicated our previous findings on gender differences in time spent in SB and different intensities of physical activity among Japanese population,^15^ through an explicit consideration of the co-dependence of time-use domains. Compared to self-report which involves reporting bias, device-based assessment can provide more accurate and reliable measures.^48^^,^^49^

Limitations of the current study should be considered. First, the Suttu town is a rural area and is not necessarily representative of Japanese cities. People in rural area may accumulate more sporadic physical activity than those in urban and suburban area.^43^ More research is needed in the different population from different geographic areas. Second, accelerometer used in this study cannot detect some types of physical activity and posture accurately. Time spent in SB and LPA may be under/overestimated in cases when participants stand still for long hours.^24^ Third, our findings may be subject to selection bias. It has been indicated that accelerometry responders are often more physically active than non-responders.^50^ In our sample, women were more likely to enroll in the accelerometer survey than men, which may affect gender differences of activity behavior patterns.

In conclusion, we demonstrated that women accumulated more LPA and less SB than men in Japanese adult population, even when time spent in other activity behaviors was taken into account. Given the health benefits of LPA, evaluating only MVPA may disproportionately underestimate the level of physical activity of women.
